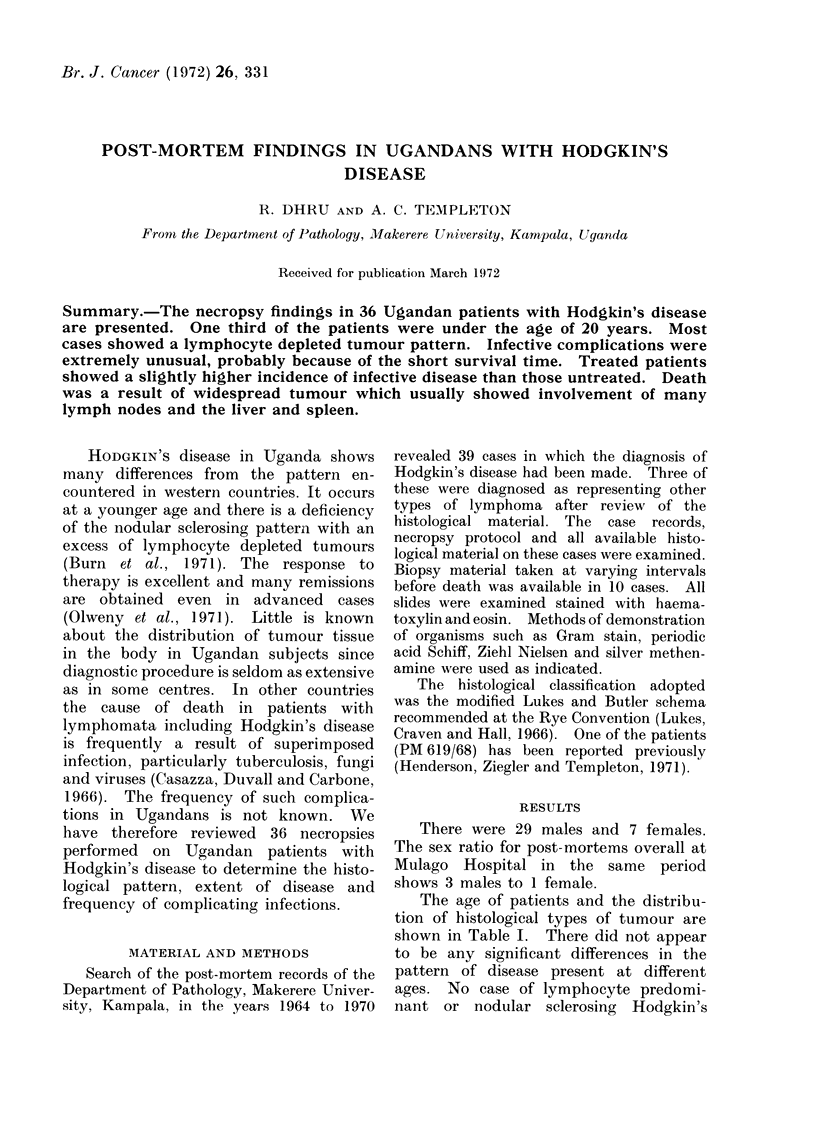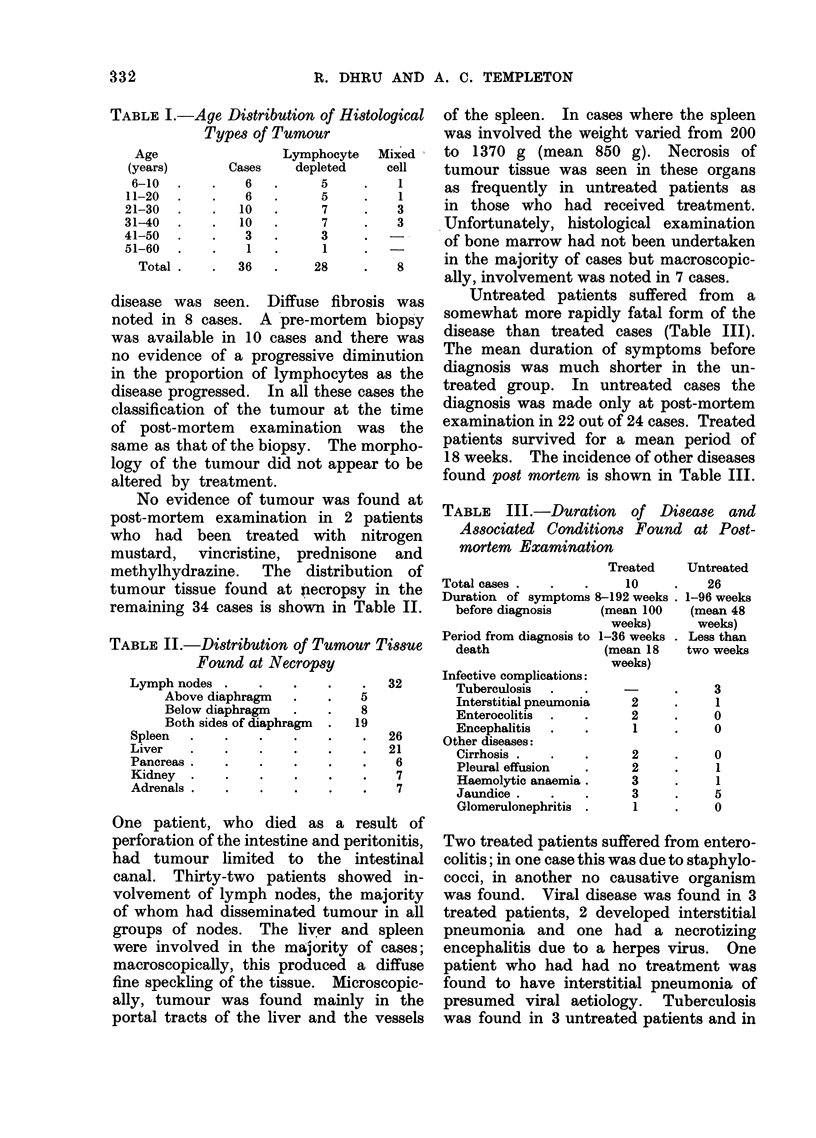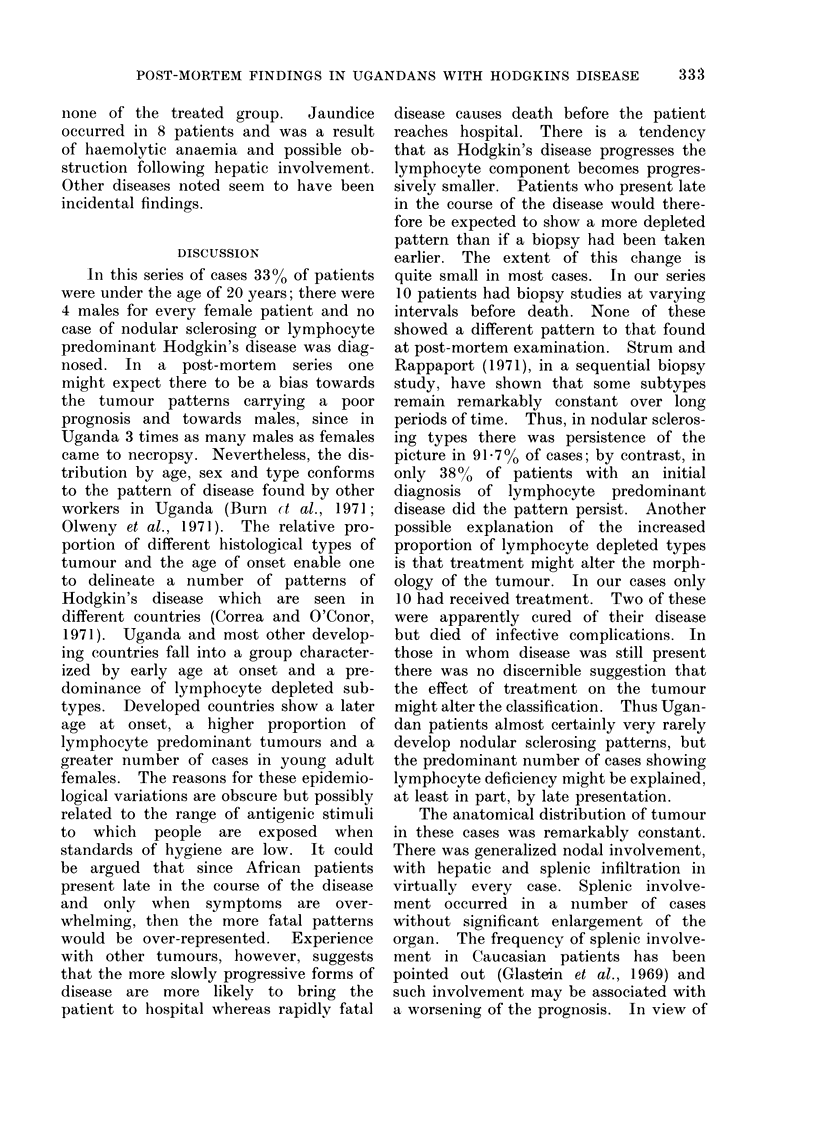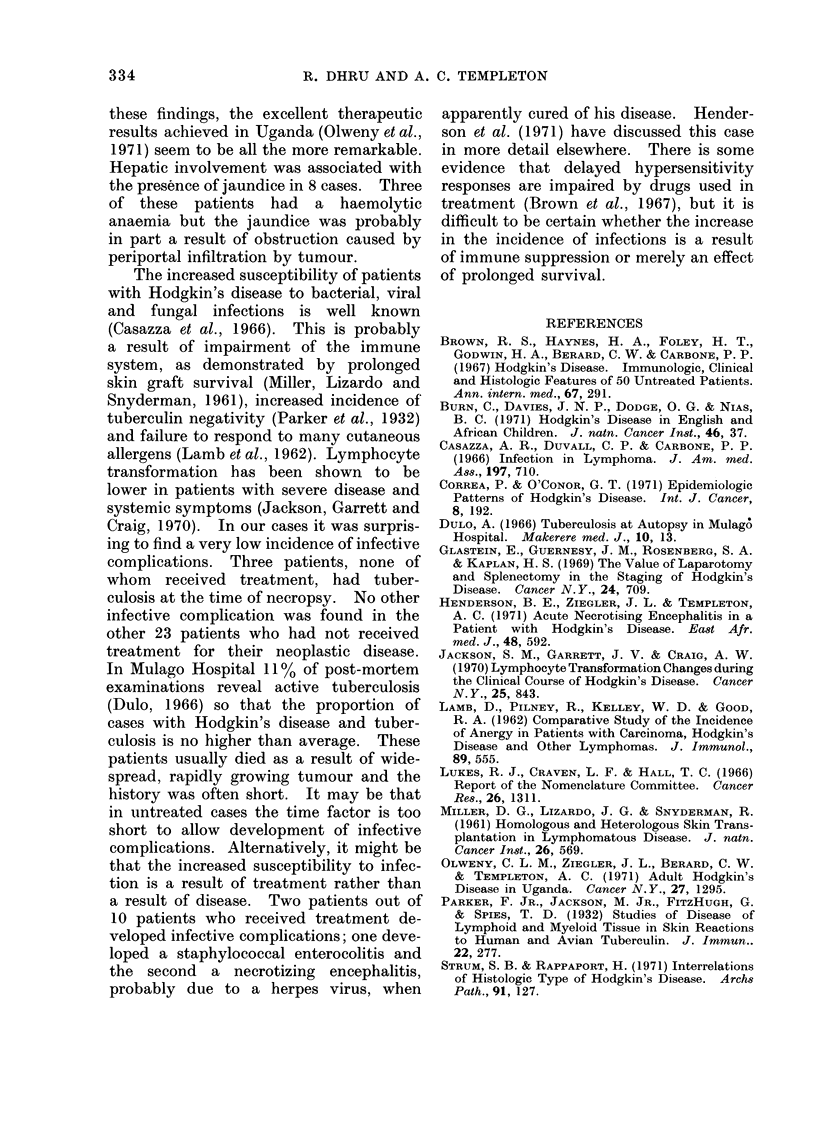# Post-mortem Findings in Ugandans with Hodgkin's Disease

**DOI:** 10.1038/bjc.1972.44

**Published:** 1972-08

**Authors:** R. Dhru, A. C. Templeton

## Abstract

The necropsy findings in 36 Ugandan patients with Hodgkin's disease are presented. One third of the patients were under the age of 20 years. Most cases showed a lymphocyte depleted tumour pattern. Infective complications were extremely unusual, probably because of the short survival time. Treated patients showed a slightly higher incidence of infective disease than those untreated. Death was a result of widespread tumour which usually showed involvement of many lymph nodes and the liver and spleen.


Br. J. Cancer (1972) **26**, 331

### POST-MORTEM FINDINGS IN UGANDANS WITH HODGKIN'S DISEASE

R. DHRU AND A. C. TEMPLETON

*From the Department of Pathology, Makerere University, Kampala, Uganda*

Received for publication March 1972

**Summary.—The necropsy findings in 36 Ugandan patients with Hodgkin's disease are presented. One third of the patients were under the age of 20 years. Most cases showed a lymphocyte depleted tumour pattern. Infective complications were extremely unusual, probably because of the short survival time. Treated patients showed a slightly higher incidence of infective disease than those untreated. Death was a result of widespread tumour which usually showed involvement of many lymph nodes and the liver and spleen.**

HODGKIN'S disease in Uganda shows many differences from the pattern encountered in western countries. It occurs at a younger age and there is a deficiency of the nodular sclerosing pattern with an excess of lymphocyte depleted tumours (Burn *et al.*, 1971). The response to therapy is excellent and many remissions are obtained even in advanced cases (Olweny *et al.*, 1971). Little is known about the distribution of tumour tissue in the body in Ugandan subjects since diagnostic procedure is seldom as extensive as in some centres. In other countries the cause of death in patients with lymphomata including Hodgkin's disease is frequently a result of superimposed infection, particularly tuberculosis, fungi and viruses (Casazza, Duvall and Carbone, 1966). The frequency of such complications in Ugandans is not known. We have therefore reviewed 36 necropsies performed on Ugandan patients with Hodgkin's disease to determine the histological pattern, extent of disease and frequency of complicating infections.

### MATERIAL AND METHODS

Search of the post-mortem records of the Department of Pathology, Makerere University, Kampala, in the years 1964 to 1970 revealed 39 cases in which the diagnosis of Hodgkin's disease had been made. Three of these were diagnosed as representing other types of lymphoma after review of the histological material. The case records, necropsy protocol and all available histological material on these cases were examined. Biopsy material taken at varying intervals before death was available in 10 cases. All slides were examined stained with haematoxylin and eosin. Methods of demonstration of organisms such as Gram stain, periodic acid Schiff, Ziehl Nielsen and silver methenamine were used as indicated.

The histological classification adopted was the modified Lukes and Butler schema recommended at the Rye Convention (Lukes, Craven and Hall, 1966). One of the patients (PM 619/68) has been reported previously (Henderson, Ziegler and Templeton, 1971).

### RESULTS

There were 29 males and 7 females. The sex ratio for post-mortems overall at Mulago Hospital in the same period shows 3 males to 1 female.

The age of patients and the distribution of histological types of tumour are shown in Table I. There did not appear to be any significant differences in the pattern of disease present at different ages. No case of lymphocyte predominant or nodular sclerosing Hodgkin's

TABLE I.—*Age Distribution of Histological Types of Tumour*

| Age (years) | Cases | Lymphocyte depleted | Mixed cell |
|---|---|---|---|
| 6–10 | 6 | 5 | 1 |
| 11–20 | 6 | 5 | 1 |
| 21–30 | 10 | 7 | 3 |
| 31–40 | 10 | 7 | 3 |
| 41–50 | 3 | 3 | — |
| 51–60 | 1 | 1 | — |
| Total | 36 | 28 | 8 |

disease was seen. Diffuse fibrosis was noted in 8 cases. A pre-mortem biopsy was available in 10 cases and there was no evidence of a progressive diminution in the proportion of lymphocytes as the disease progressed. In all these cases the classification of the tumour at the time of post-mortem examination was the same as that of the biopsy. The morphology of the tumour did not appear to be altered by treatment.

No evidence of tumour was found at post-mortem examination in 2 patients who had been treated with nitrogen mustard, vincristine, prednisone and methylhydrazine. The distribution of tumour tissue found at necropsy in the remaining 34 cases is shown in Table II.

TABLE II.—*Distribution of Tumour Tissue Found at Necropsy*

| | | |
|---|---|---|
| Lymph nodes | | 32 |
| Above diaphragm | 5 | |
| Below diaphragm | 8 | |
| Both sides of diaphragm | 19 | |
| Spleen | | 26 |
| Liver | | 21 |
| Pancreas | | 6 |
| Kidney | | 7 |
| Adrenals | | 7 |

One patient, who died as a result of perforation of the intestine and peritonitis, had tumour limited to the intestinal canal. Thirty-two patients showed involvement of lymph nodes, the majority of whom had disseminated tumour in all groups of nodes. The liver and spleen were involved in the majority of cases; macroscopically, this produced a diffuse fine speckling of the tissue. Microscopically, tumour was found mainly in the portal tracts of the liver and the vessels of the spleen. In cases where the spleen was involved the weight varied from 200 to 1370 g (mean 850 g). Necrosis of tumour tissue was seen in these organs as frequently in untreated patients as in those who had received treatment. Unfortunately, histological examination of bone marrow had not been undertaken in the majority of cases but macroscopically, involvement was noted in 7 cases.

Untreated patients suffered from a somewhat more rapidly fatal form of the disease than treated cases (Table III). The mean duration of symptoms before diagnosis was much shorter in the untreated group. In untreated cases the diagnosis was made only at post-mortem examination in 22 out of 24 cases. Treated patients survived for a mean period of 18 weeks. The incidence of other diseases found *post mortem* is shown in Table III.

TABLE III.—*Duration of Disease and Associated Conditions Found at Post-mortem Examination*

| | Treated | Untreated |
|---|---|---|
| Total cases | 10 | 26 |
| Duration of symptoms before diagnosis | 8–192 weeks (mean 100 weeks) | 1–96 weeks (mean 48 weeks) |
| Period from diagnosis to death | 1–36 weeks (mean 18 weeks) | Less than two weeks |
| Infective complications: | | |
| Tuberculosis | — | 3 |
| Interstitial pneumonia | 2 | 1 |
| Enterocolitis | 2 | 0 |
| Encephalitis | 1 | 0 |
| Other diseases: | | |
| Cirrhosis | 2 | 0 |
| Pleural effusion | 2 | 1 |
| Haemolytic anaemia | 3 | 1 |
| Jaundice | 3 | 5 |
| Glomerulonephritis | 1 | 0 |

Two treated patients suffered from enterocolitis; in one case this was due to staphylococci, in another no causative organism was found. Viral disease was found in 3 treated patients, 2 developed interstitial pneumonia and one had a necrotizing encephalitis due to a herpes virus. One patient who had had no treatment was found to have interstitial pneumonia of presumed viral aetiology. Tuberculosis was found in 3 untreated patients and in none of the treated group. Jaundice occurred in 8 patients and was a result of haemolytic anaemia and possible obstruction following hepatic involvement. Other diseases noted seem to have been incidental findings.

### DISCUSSION

In this series of cases 33% of patients were under the age of 20 years; there were 4 males for every female patient and no case of nodular sclerosing or lymphocyte predominant Hodgkin's disease was diagnosed. In a post-mortem series one might expect there to be a bias towards the tumour patterns carrying a poor prognosis and towards males, since in Uganda 3 times as many males as females came to necropsy. Nevertheless, the distribution by age, sex and type conforms to the pattern of disease found by other workers in Uganda (Burn *et al.*, 1971; Olweny *et al.*, 1971). The relative proportion of different histological types of tumour and the age of onset enable one to delineate a number of patterns of Hodgkin's disease which are seen in different countries (Correa and O'Conor, 1971). Uganda and most other developing countries fall into a group characterized by early age at onset and a predominance of lymphocyte depleted subtypes. Developed countries show a later age at onset, a higher proportion of lymphocyte predominant tumours and a greater number of cases in young adult females. The reasons for these epidemiological variations are obscure but possibly related to the range of antigenic stimuli to which people are exposed when standards of hygiene are low. It could be argued that since African patients present late in the course of the disease and only when symptoms are overwhelming, then the more fatal patterns would be over-represented. Experience with other tumours, however, suggests that the more slowly progressive forms of disease are more likely to bring the patient to hospital whereas rapidly fatal disease causes death before the patient reaches hospital. There is a tendency that as Hodgkin's disease progresses the lymphocyte component becomes progressively smaller. Patients who present late in the course of the disease would therefore be expected to show a more depleted pattern than if a biopsy had been taken earlier. The extent of this change is quite small in most cases. In our series 10 patients had biopsy studies at varying intervals before death. None of these showed a different pattern to that found at post-mortem examination. Strum and Rappaport (1971), in a sequential biopsy study, have shown that some subtypes remain remarkably constant over long periods of time. Thus, in nodular sclerosing types there was persistence of the picture in 91·7% of cases; by contrast, in only 38% of patients with an initial diagnosis of lymphocyte predominant disease did the pattern persist. Another possible explanation of the increased proportion of lymphocyte depleted types is that treatment might alter the morphology of the tumour. In our cases only 10 had received treatment. Two of these were apparently cured of their disease but died of infective complications. In those in whom disease was still present there was no discernible suggestion that the effect of treatment on the tumour might alter the classification. Thus Ugandan patients almost certainly very rarely develop nodular sclerosing patterns, but the predominant number of cases showing lymphocyte deficiency might be explained, at least in part, by late presentation.

The anatomical distribution of tumour in these cases was remarkably constant. There was generalized nodal involvement, with hepatic and splenic infiltration in virtually every case. Splenic involvement occurred in a number of cases without significant enlargement of the organ. The frequency of splenic involvement in Caucasian patients has been pointed out (Glastein *et al.*, 1969) and such involvement may be associated with a worsening of the prognosis. In view of these findings, the excellent therapeutic results achieved in Uganda (Olweny *et al.*, 1971) seem to be all the more remarkable. Hepatic involvement was associated with the presence of jaundice in 8 cases. Three of these patients had a haemolytic anaemia but the jaundice was probably in part a result of obstruction caused by periportal infiltration by tumour.

The increased susceptibility of patients with Hodgkin's disease to bacterial, viral and fungal infections is well known (Casazza *et al.*, 1966). This is probably a result of impairment of the immune system, as demonstrated by prolonged skin graft survival (Miller, Lizardo and Snyderman, 1961), increased incidence of tuberculin negativity (Parker *et al.*, 1932) and failure to respond to many cutaneous allergens (Lamb *et al.*, 1962). Lymphocyte transformation has been shown to be lower in patients with severe disease and systemic symptoms (Jackson, Garrett and Craig, 1970). In our cases it was surprising to find a very low incidence of infective complications. Three patients, none of whom received treatment, had tuberculosis at the time of necropsy. No other infective complication was found in the other 23 patients who had not received treatment for their neoplastic disease. In Mulago Hospital 11% of post-mortem examinations reveal active tuberculosis (Dulo, 1966) so that the proportion of cases with Hodgkin's disease and tuberculosis is no higher than average. These patients usually died as a result of widespread, rapidly growing tumour and the history was often short. It may be that in untreated cases the time factor is too short to allow development of infective complications. Alternatively, it might be that the increased susceptibility to infection is a result of treatment rather than a result of disease. Two patients out of 10 patients who received treatment developed infective complications; one developed a staphylococcal enterocolitis and the second a necrotizing encephalitis, probably due to a herpes virus, when apparently cured of his disease. Henderson *et al.* (1971) have discussed this case in more detail elsewhere. There is some evidence that delayed hypersensitivity responses are impaired by drugs used in treatment (Brown *et al.*, 1967), but it is difficult to be certain whether the increase in the incidence of infections is a result of immune suppression or merely an effect of prolonged survival.

### REFERENCES

BROWN, R. S., HAYNES, H. A., FOLEY, H. T., GODWIN, H. A., BERARD, C. W. & CARBONE, P. P. (1967) Hodgkin's Disease. Immunologic, Clinical and Histologic Features of 50 Untreated Patients. *Ann. intern. med.*, **67**, 291.

BURN, C., DAVIES, J. N. P., DODGE, O. G. & NIAS, B. C. (1971) Hodgkin's Disease in English and African Children. *J. natn. Cancer Inst.*, **46**, 37.

CASAZZA, A. R., DUVALL, C. P. & CARBONE, P. P. (1966) Infection in Lymphoma. *J. Am. med. Ass.*, **197**, 710.

CORREA, P. & O'CONOR, G. T. (1971) Epidemiologic Patterns of Hodgkin's Disease. *Int. J. Cancer*, **8**, 192.

DULO, A. (1966) Tuberculosis at Autopsy in Mulago Hospital. *Makerere med. J.*, **10**, 13.

GLASTEIN, E., GUERNESY, J. M., ROSENBERG, S. A. & KAPLAN, H. S. (1969) The Value of Laparotomy and Splenectomy in the Staging of Hodgkin's Disease. *Cancer N.Y.*, **24**, 709.

HENDERSON, B. E., ZIEGLER, J. L. & TEMPLETON, A. C. (1971) Acute Necrotising Encephalitis in a Patient with Hodgkin's Disease. *East Afr. med. J.*, **48**, 592.

JACKSON, S. M., GARRETT, J. V. & CRAIG, A. W. (1970) Lymphocyte Transformation Changes during the Clinical Course of Hodgkin's Disease. *Cancer N.Y.*, **25**, 843.

LAMB, D., PILNEY, R., KELLEY, W. D. & GOOD, R. A. (1962) Comparative Study of the Incidence of Anergy in Patients with Carcinoma, Hodgkin's Disease and Other Lymphomas. *J. Immunol.*, **89**, 555.

LUKES, R. J., CRAVEN, L. F. & HALL, T. C. (1966) Report of the Nomenclature Committee. *Cancer Res.*, **26**, 1311.

MILLER, D. G., LIZARDO, J. G. & SNYDERMAN, R. (1961) Homologous and Heterologous Skin Transplantation in Lymphomatous Disease. *J. natn. Cancer Inst.*, **26**, 569.

OLWENY, C. L. M., ZIEGLER, J. L., BERARD, C. W. & TEMPLETON, A. C. (1971) Adult Hodgkin's Disease in Uganda. *Cancer N.Y.*, **27**, 1295.

PARKER, F. JR., JACKSON, M. JR., FITZHUGH, G. & SPIES, T. D. (1932) Studies of Disease of Lymphoid and Myeloid Tissue in Skin Reactions to Human and Avian Tuberculin. *J. Immun.*, **22**, 277.

STRUM, S. B. & RAPPAPORT, H. (1971) Interrelations of Histologic Type of Hodgkin's Disease. *Archs Path.*, **91**, 127.